# An analysis of the effects of a campaign supporting use of a health symbol on food sales and shopping behaviour of consumers

**DOI:** 10.1186/s12889-017-4149-3

**Published:** 2017-03-09

**Authors:** Trine Mørk, Klaus G. Grunert, Morten Fenger, Hans Jørn Juhl, George Tsalis

**Affiliations:** MAPP Centre, Aarhus University, Bartholins Alle 14, 8000 Aarhus C, Denmark

**Keywords:** Food choice, Health symbol, Health campaign, Transaction data, Store interview

## Abstract

**Background:**

Since 2009, the green Keyhole symbol has been a joint Nordic initiative for signalling healthfulness of specific food products. In 2014, the Danish Ministry of Food, Agriculture and Fisheries carried out a campaign aimed mainly at men over 35 with a low level of education, encouraging them to use the Keyhole in their shopping process. The objective of the study is to evaluate the campaign by measuring its effect on consumer behaviour in the store.

**Methods:**

The impact of the Keyhole campaign was measured in selected retail stores. Sales data were analysed to ascertain whether sales of Keyhole labelled products changed during and after the campaign. Observations and interviews were conducted in the same stores.

**Results:**

The campaign had a positive effect on sales of Keyhole-labelled products in two out of three retail chains investigated. In these two retail chains, sales of Keyhole labelled products rose by about 20%. In the third chain, there was a slight decrease of sales of Keyhole labelled products. The effect differed considerably between product categories. Analysis of the interview data indicated that by the end of the campaign, shoppers with a short education had a higher likelihood of mentioning health as a purchase motive, and there was a higher general tendency to look for nutrition information.

**Conclusions:**

Results suggest that the campaign did have effects on shopper behaviour and that it is possible to address shoppers with a short education by a tailored campaign. However, long-term effect of the campaign was not ascertained.

## Background

In order to encourage healthier food choices, a variety of nutrition labelling schemes, both compulsory and voluntary, are employed to signal healthier alternatives when making choices. Different types of nutrition labelling schemes have been investigated for their effect on consumers (for reviews see [1–4]) with mixed results, suggesting that while many consumers understand such information and can make adequate use of it when prompted to do so [5], lack of motivation to use the information and lack of attention to it in real-life store environments are major bottlenecks regarding effects on actual food choice [6]. The habitual and heuristic nature of many food choices [7] suggests that simplified and/or symbolic nutrition information may be a promising way to encourage healthier food choices. This includes health symbols, i.e., symbols on the package or the shelf front that designate food products as a healthier choice without invoking numerical information on calories or nutrients. Examples of health symbols are the Heart symbol in the U.S and Finland, the Keyhole in Norway, Sweden, Iceland and Denmark, the Choices logo in Holland, Belgium, Poland, Czech Republic, and Slovakia and The Heart Foundation Tick from New Zealand.

Health symbols are often not self-explanatory, and organizations sponsoring health symbols, both state-sponsored and otherwise, employ a range of measures to promote consumer awareness, understanding, and use. This can take many forms, including mass media advertising, TV commercials, posters in stores, sweepstakes, and web-based information. Whether such measures indeed have an effect on consumers’ use of health symbols is an underresearched topic. The mixed results of studies investigating the use and effects of health symbols (e.g., [8–10]) may be, in part, related to differences in the way and extent to which these symbols were supported by measures to promote awareness, understanding and use of such symbols. Research on the effects of such support measures could therefore be a valuable contribution to our knowledge on the effects (or lack of same) of health symbols aimed at promoting healthier choices.

In the present paper, we analyse the effects of a multi-media campaign that the Danish Ministry of Food, Agriculture and Fisheries carried out in 2014 to promote the Nordic Keyhole health symbol. The Keyhole is a voluntary health symbol, the purpose of which is to highlight a “better choice” of food products within a product category. The Keyhole is awarded if the product meets the criteria established by the government in a nutrient profile model. Each year, The Ministry of Food, Agriculture and Fisheries in Denmark runs a campaign, usually for a period of 4 weeks, to raise awareness about the Keyhole and to promote the use of the label among consumers. Danish consumers are aware of the Keyhole symbol, but have limited knowledge about what the Keyhole label stands for [11]. The 2014 campaign is especially interesting because it, while addressing the general audience, also had a more narrowly defined target group: Men older than 35 with a low level of education. It is an often-voiced concern that the incidence of unhealthy dietary patterns is related to socio-economic class [12, 13]. For example, in Denmark – a traditionally egalitarian country – the highest share of obese is among citizens with low socio-economic status [14]. In a recent national study researchers found that 18.6% of Danish men have an unhealthy dietary pattern (based on a diet score based system calculated on the basis of self-reported consumption, where respondents were categorized as having an unhealthy dietary pattern if they report a low intake of fruit, vegetables and fish and a high consumption of saturated fatty acids). For women the percentage is 9.5%. When education is taken into account, the study finds that 4.6% of those who completed a higher education have an unhealthy dietary pattern compared to 24.8% of those who did not [15].

In this paper, we will provide evidence on some effects of the Keyhole campaign 2014. The knowledge obtained from the study has implications both for the design of future campaigns and for the potential of health symbols to affect consumer choice. We will start by outlining the theoretical framework of the study before explaining the methodology. Following this, we will present and discuss the results. We will conclude the article by outlining the significance of the results for promoting the use of health symbols.

## Theoretical framework

In this study, we view a consumer’s choice of a food product as the result of a process that is somewhere between a deliberate, consciously formed decision and a habitual purchase. Habitually driven decisions are repetitions of earlier decisions in the same environment [16] and may ultimately mean that a product is purchased purely based on product recognition. The more a choice resembles a deliberate decision, the more information processing will be involved, in-store and/or by previous exposure to product-related information. However, in most cases, even deliberate decisions will be simplified decisions that are characterized by the use of heuristics - simple decision rules - and thus only limited processing of information will be involved, especially in the shop, where decisions are usually made quickly [17, 18]. Heuristics involve the use of a few key criteria such as brand and price, but possibly also criteria related to health and convenience. Heuristics can be modified depending on the decision situation, including the activation of purchase motives at the time of decision [19].

A campaign that aims to promote the use of the Keyhole can influence consumer choice in several ways. First, consumers, especially those with a high health consciousness, may already have the Keyhole as part of their repertoire of heuristics, and a campaign has the potential to increase the frequency of its use by priming it and thus making it more accessible in the purchase situation. Second, consumers who do not usually use health-related heuristics or use other health-related heuristics can be encouraged to use the Keyhole in their attempts to find healthy alternatives. Finally, a campaign could increase the presence of health motivation in the purchasing process, which may affect whether or not the Keyhole is used as information in this process.

The possibilities of influencing consumers in the store are limited, because the amount of information is overwhelming and consumers spend only little time in making decisions. Possible promotional effects are likely to be based on the combined effects of multiple information channels. Information channels that work outside the shops can both lead to learning new information (e.g. about the meaning of the Keyhole) and the prioritization of health motives, which in turn can increase the likelihood that health-related information is used both inside and outside the store.

Research indicates that health symbols, such as the Keyhole, can increase, decrease, or have no effect on the sales of the products that bear them [8–10, 20]. However, studies on the effects of health symbols have not usually taken into account to which extent campaigns have been used to introduce and/or promote the symbol. This may be one reason for the diversity of results reported in previous studies.

## Methods

### Context

In 2014, the Danish Ministry of Food, Agriculture and Fisheries carried out a 3-week campaign promoting the use of the Keyhole health symbol. The campaign was especially directed at men over 35 years of age with 9–10 years of school, with or without vocational training, but without further education. The Ministry formed an alliance with three major retail chains that received promotional material to decorate the stores, materials for competitions with prizes for both employees and customers, and several recipes that were developed together with a well-known TV chef specifically to appeal to the special target group. These were available in the participating stores and on the specially designed Keyhole website. In-store promotional activities consisted of ceiling signs, tattoos with the keyhole on, posters featuring the TV chef and a large knife, flyers with reference to what the keyhole is based on and shelf labels also encouraging participation in a sweepstake (see Table 1). Other activities in-store were promotional videos in the fruit and vegetables section or at the counter with fresh fish, showing the TV chef creating one of the special designed meals for the campaign. Outside the store environment, radio spots, TV spots, and special events ran throughout the campaign period.

**Table 1:** Examples of campaign material

Campaign material	Design
Ceiling signs	
Tattoos	
Posters	
Commercial flyers	
Shelf labels	

Reproduced with permission from the Danish Food and Veterinary Administration

### Overview of data collection

The impact of the Keyhole campaign was measured in selected retail stores. The study is in two parts. The first part consists of the analysis of sales data in selected stores, analysing whether sales of Keyhole labelled products changed during and after the campaign. These data were provided by the participating retail chains. The second part consists of observations and interviews in selected stores. While the analysis of sales data does not allow differentiating the effects by different target groups, the observation/interview study allows us to distinguish effects on different groups of shoppers, including the target group of the campaign.

### Selection of stores

Six retail shops belonging to three different retail chains were selected based on nominations from the participating retail chains. A criterion for the selection of stores was that the campaign's focal consumer group - men over 35 years of age with limited education – had a high probability of shopping there. Likelihood of the focal consumer group shopping there was assessed based on store location and store managers’ indication that the target group was shopping there. For practical reasons, all six stores also had to be within a reasonable geographical distance from each other. Chains A and C were typical full-assortment supermarkets, whereas chain B was a discount chain with a smaller assortment. The chains differed in the share of Keyhole-labelled products in the assortment. Use of the campaign material was monitored during the three campaign weeks in each store on a daily basis, using a checklist. No systematic differences in the use of the campaign material were detected between the different stores.

### Analysis of sales data

The sales data analyses were limited to the following product categories: Fresh fruit and vegetables, frozen fruit and vegetables, frozen ready meals, fresh fish, frozen fish, fresh meat, frozen meat, flavoured fermented milk products, breakfast cereals, and pre-packed bread. The selection of these categories was based on the assumption that they were likely to contain products labelled with the Keyhole logo, while also having some variation in the share of products that would be eligible for the Keyhole.

For each of the above categories, sales data were obtained on a daily basis for all days in the following weeks in 2014: 14, 15, 17 (week 16 omitted due to Easter holidays), weeks 18, 19, 20 (Keyhole campaign), and weeks 21, 22, 23. Data obtained were total turnover in terms of both volume and value per category, total turnover in terms of both volume and value for Keyhole labelled products in category, number of transactions in the product category, number of total transactions for Keyhole labelled products and information about average price of each product, all on a daily basis, resulting in 9 × 7 = 63 data points..

### In-store observation and interview

The second part consisted of observations and subsequent interviews with customers in the selected stores. This part of the study was carried out in four out of the six stores used in part 1 of the study, those from retail chains A and B. It was not possible to carry out interviews in retail chain C due to resource constraints and delays in getting the necessary approval.

Shoppers were observed and subsequently approached for a short interview at the shelf with breakfast cereals and at the counter with ready meals. Our decision to concentrate on breakfast cereals and ready meals was based on the assumption that the breakfast cereals category covers a wide variety of products, both more and less healthful [21, 22]. Furthermore, the special target group was assumed to be frequent consumers of ready meals. This assumption is corroborated by studies linking men to be more frequent consumers of ready meals, in part because of less developed cooking skills and a more positive attitude towards the product category [23, 24]. In both categories, some products carried the Keyhole while others did not. Chain A had fewer Keyhole labelled products on its shelves, 459, than chain B, which had 961.

During the observations, we noted whether the customers looked at the product and, if so, whether they looked at the front or other areas of the product. We also noted whether they observed the campaign-related material in the store and how long it took them to select the product. If a selection took place, we asked the shopper to participate in an interview. Observational data from non-consenting shoppers were deleted. The interview employed a questionnaire gathering data about age, employment, educational background, which item they had just selected, if they had purchased that item before (yes/no/can’t remember), why they chose this item (open question), if they had looked for nutritional information (yes/no/can’t remember) and if yes, if they had looked for any labels on the product (open question, but to be confirmed by respondent by showing a picture of the label). If the customer indicated that s/he had looked for the Keyhole, s/he asked to show where they found the symbol, if they knew what the Keyhole means (open question), how often they look for the Keyhole when shopping (never/rarely/often/always), if they have noticed any special Keyhole activities in the store or on the flyers (open question) and how much time they had to shop for groceries that day (little/appropriate/plenty). Answers to the open questions were coded afterwards.

In total, 1411 respondents were recruited in the four retail stores. All respondents had to be 18 years of age or older. Half of the respondents were men over 35 years of age with between 9 and 10 years of school, with or without vocational training, but without further education. Half of the respondents were recruited at the cereal shelf, and the other half at the counter with ready meals. Half of the respondents were recruited and interviewed before the Keyhole campaign in week 17 and the other half towards the end of the Keyhole campaign in week 20.

## Results

### Analysis of sales data

**Fig. 1:**
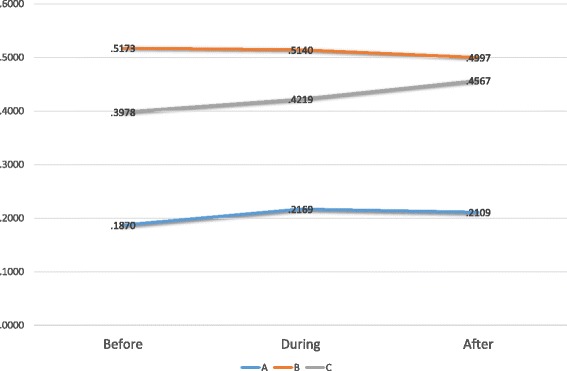
Share of transactions with Keyhole labelled products by retail chain

Figure 1 shows the mean share of transactions with Keyhole labelled products across all product categories in the dataset for the three retail chains before, during and after the campaign. The data show that the share of transactions with Keyhole labelled products differs considerably between the three retail chains, with discount chain B having the highest share. Share of transactions with Keyhole labelled products decreased slightly during the campaign for chain B, increased during the campaign for chains A and C, and levelled off after the campaign for chain A, while it continued to raise for chain C. These figures, however, cover strong variations between product categories. Some product categories, most notably fresh and frozen fish, but also cereals, show a strong increase, whereas other product categories remain almost constant or even decrease.

Because of the different context in the three retail chains, statistical analysis was done separately for three chains. For each, chain, a multi-level logistic regression was estimated in SAS with the logit of the share of keyhole labelled products per day and per product category as the dependent variable. Time period (during/post campaign vs. pre campaign) and the relative price differential of Keyhole labelled products compared to the remaining products in the category were estimated as fixed effect parameters. Product category was specified as a random factor. The results are shown in Table 2. For all three retail chains, the price differential for Keyhole labelled products affects the odds for buying Keyhole labelled products. For retail chains A and C, the odds for buying Keyhole labelled products rises by about 20% during the campaign period, and this effect is sustained in the 3 weeks after the campaign. For the discount chain B, where the share of transactions with Keyhole labelled products was highest before the campaign, the odds for buying Keyhole products decreased by about 10% during the campaign, and again a negative effect remained in the 3 weeks after the campaign. All effects are statistically significant. For additional illustration, Table 3 shows total number of transactions and total value for Keyhole-labelled and not Keyhole-labelled products for the three retail chains pre-, during and post-campaign. Again, the results indicate considerable differences between the three retail chains. In the discount chain B, most of the transactions and the bigger share of the total value relates to products with the keyhole. This may be partly due to the smaller assortment of the chain, which includes a larger share of staple and a smaller share of specialty products.

**Table 2:** Share of Keyhole labelled products as explained by campaign period and price

Predictor		Coefficient	p	Odds ratio	Coefficient	*p*	Odds ratio	Coefficient	*p*	Odds ratio
		Retail chain A			Retail chain B			Retail chain C		
Fixed effects										
Intercept		−1.091	.03		1.230	.04		.594	.02	
Relative price		−.955	.00		−1.129	.00		−1.216	.00	
During campaign		.186	.00	1.204	−.114	.00	0.892	.175	.00	1.191
Post campaign		.162	.00	1.176	−.219	.00	0.803	.283	.00	1.327
Random effects										
Bread	Intercept	−2.457	.00		.513	.29		−.012	.78	
Cereals	Intercept	1.700	.00		.004	.99		.544	.20	
Fresh fish	Intercept	1.900	.00		1.428	.00		1.831	.00	
Frozen fish	Intercept	1.302	.00		.513	.30		.029	.94	
Fresh meat	Intercept	−2.351	.00		−.863	.08		−1.964	.00	
Frozen meat	Intercept							−.718	.10	
Fresh greens	Intercept	.903	.04		1.670	.00		−.439	.30	
Frozen greens	Intercept	.582	.18		1.183	.01		2.011	.00	
Frozen ready meals	Intercept	−1.268	.00		−2.271	.00		−1.879	.00	
Chilled ready meals	Intercept	−1.216	.00							
Sour milk products	Intercept	−.205	.64		−2.177	.00		.701	.10

**Table 3:**
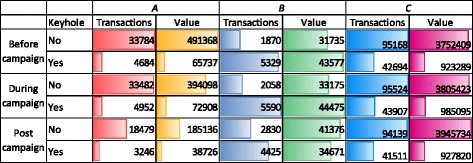
Average daily number of transactions and volume of transactions w/o Keyhole before, during and after campaign (value in in Danish kroner, numbers aggregated over selected product categories)

In order to provide additional support to the conclusion that the differences between the periods are related to the campaign and not just to seasonal fluctuation, we obtained, for retail chain A, the same data for the same periods the year before the campaign and estimated the same model. The results indicated no significant effect for the same periods.

### Analysis of store observations and interviews

Data from a total of 1411 respondents were gathered: 602 respondents within the focal consumer segment of men over 35 years with low education, 528 respondents with an advanced educational background, and 281 other respondents (e.g., not male or with age <35) with short/without education. Demographics characteristics of the sample can be seen in Table 4.

**Table 4:** Demographic characteristics by shopper segment

	Segment 1: Longer education	Segment 2: Short education, but not men > 35 years	Segment 3: Men > 35 years with short eduction (special target group)
Age			
18–35	29.0%	63.7%	0%
36–44	19.9%	5.7%	47.7%
45–54	22.0%	6.8%	18.3%
55–64	18.2%	9.3%	16.3%
65 +	11.0%	14.6%	17.8%
Gender			
Male	44.3%	32.4%	100.0%
Female	55.7%	67.6%	0%
Highest level of education			
Primary school	0%	11.4%	14.3%
Secondary school	0%	46.6%	15.3%
Vocational	0%	42.0%	70.4%
Higher education	100.0%	0%	0%
N	528	281	602

ANOVAs and logistic regressions were estimated to ascertain the effect of the campaign as opposed to the pre-campaign period for the following dependent variables: 1) the time it took consumers to decide on their purchases, 2) the probability that the consumers looked at the front of the product’s package (the location of the Keyhole), 3) whether health was a motive mentioned by consumers as a reason for their choice in the corresponding open-ended question in the interview 4) the probability that respondents looked for nutritional information, and 5) the probability that respondents looked for the Keyhole logo. Descriptive statistics for these measures can be seen in Table 5. In all cases, the analysis controlled for product category and retail chain, and consumer segment. In order to emphasize possible differences between the three consumer segments (men > 35 with a short education, the special target group of the campaign; other shoppers with a short education; shoppers with a long education), three-class models were estimated in LatentGold. When the Wald statistic indicated that there were no significant differences between the three segments (*p* > .1), coefficients for the three segments were constrained to be equal. There was no campaign effect regarding the time used to make a purchase decision (mean time was 21.8 s, SD = 23.1). Likewise, there was no significant campaign effect on the likelihood to look at the front of the package, although the likelihood differed by retail chain.

**Table 5:** Measures for in-store behaviour

		Before the Keyhole campaign	During the Keyhole campaign
Time until purchase (sec)	Mean	21.73	21.92
Looked at front of pack	no	15.7%	13.7%
	yes	84.3%	86.3%
Health as a purchase motive	no	81.9%	78.5%
	yes	18.1%	21.5%
Looked at nutrition info	no	73.7%	62.9%
	yes	26.3%	37.1%
Look for Keyhole (only shoppers who looked for nutrition info)	no	43.3%	31.8%
	yes	56.7%	68.2%
Total N		708	703

The likelihood of mentioning ‘health’ as one of the purchase motives for the purchase just made was affected by the campaign (see Table 6). Likelihood of mentioning health as a purchase motive was almost double in the campaign week as opposed to before the campaign for those respondents who had short education but were not part of the special target group. It also increased for the special target group, while it slightly decreased for respondents with a longer education. The likelihood was smaller for ready meals as compared to cereals, and in the discount chain B as compared to chain A. These effects did not differ between the three segments.

**Table 6:** Effect of campaign on purchase behaviour in store

Dependent: Health as one of the purchase motives								
	Class 1: Longer education		Class 2: Short education, not men >35 years		Class 3: Men > 35 years with short education		p* (main effect)	p* (difference between classes)
	Coefficient	Odds ratio	Coefficient	Odds ratio	Coefficient	Odds ratio		
Intercept	−0.755		−1.362		−1.094		.00	.07
Period: Campaign (vs. pre-campaign)	−0.215	.806	0.749	2.114	0.247	1.280	.00	.03
	Coefficient			Odds ratio				
Product: Ready meals (vs. cereals)	−0.698			.497			.00	
Retail chain: B (vs. A)	−0.398			.671			.00	
Dependent: Looked for nutrition information								
Intercept	−.944							
Period: Campaign (vs. pre-campaign)	.469			1.598			.00	
Product: Ready meals (vs. cereals)	ns							
Retail chain: B (vs. A)	.286			1.331			.01	

*Wald statistic

The likelihood that one looked for nutrition information on the package before making the purchase was likewise different pre/during campaign. At the end of the campaign, this likelihood was 60% higher than before the campaign. This effect did not differ between segments. The likelihood was also higher in retail chain B.

For those shoppers who answered that they did look for nutrition information, it was additionally asked whether they had looked for the Keyhole. This probability was not affected pre/during campaign.

## Discussion

The choices made in grocery stores every day are important for the health of the general population. The introduction of health symbols has the aim to further healthy choices. However, research on the effects of such symbols on food choice has had mixed results. One of the reasons for this may be that health symbols need to be supported by campaigns promoting awareness, understanding and use of the symbol, and the effects of such campaigns have not been subject to scientific scrutiny. In this study, we analysed the effects of a campaign aimed at promoting the use of the Keyhole symbol in Denmark, a campaign that was specifically aimed at the target group men > 35 with a short education. In order to detect possible campaign effects, we collected sales data from six retail stores from three different retail chains and analysed the share of sales of products with a Keyhole before, during and after the campaign. Our results suggest that the Keyhole campaign had an impact on the share of sales of Keyhole-labelled products. The effect during and after the campaign varied across the retail chains: The retail chain with the lowest share of Keyhole-labelled products showed an increase in the sales of those products during the campaign which continued after the completion of the campaign, whereas the retail chain with the highest share of Keyhole-labelled products actually experienced a drop of sales of Keyhole-labelled products during the same period.

We can only speculate about the reasons for the decline of sales of Keyhole labelled product in chain B. As noted, this was the chain with the highest share of transactions with Keyhole labelled products in the pre-campaign period. At the same time, it is (in contrast to chains A and B) a discount chain and may attract fewer health-conscious shoppers, which is supported by the finding that the likelihood of shoppers there having had health as a shopping motive was considerably lower compared to chain A. The campaign may, for some shoppers in chain B, have had the effect of alerting them to the presence of the Keyhole on many products in these shops and may have resulted in actual avoidance behaviour. Since the Keyhole was more prominent in chain B compared to chains A and C, avoidance behaviour would be easier to implement there, with a decrease in transactions involving Keyhole labelled products as a result. Recent results from an eye-tracking study suggest indeed that consumers from our focal target group (men > 35 with a short education) may try to avoid looking at the Keyhole when making choices [25].

To substantiate the results from the analysis of the transaction data, we carried out observations and interviews in four out of the six stores. 1411 store interviews were carried out before and towards the end of the campaign in order to analyse changes in shopping behaviour with regard to two selected product categories. Results indicated some increase in having health as a shopping motive and in looking for nutrition information in general, though not in looking specifically for the Keyhole. The effect on having health as a shopping motive was limited to shoppers having a short education, which may be due to the fact that their likelihood of having health as a shopping motive is lower in the first place. The effect on looking for nutrition information did not differ significantly between consumer segments.

The campaign studied here used a range of different materials, including some to be used in-store. While promotional material to be used in the shops was a major element of the Keyhole campaign, it turned out that actual use of this material in the shops where the study was carried out was limited. This makes it unlikely that the effects came about only as a result of what happened in the stores. On the contrary, it is more likely that the measured effects are the result of the multi-media campaign, in which different communication channels worked together. Many successful social marketing campaigns with different aims acknowledge the cumulative effects of multi-channel communication [26], which are also in line with the increasingly more common practice of multichannel retailing [27].

Several limitations of the study apply. First, the results are based on analysis of a few selected stores. Second, the observation/interview part of the study is limited by the selection of product categories. The frozen ready meal counter was not very well visited and might be one of the categories where a nutrition label could even have a reverse effect. Thirdly, the studies were carried out within a limited time frame, which means that we did not measure the long-term effect of the campaign. Though the sales data covered a period of 3 weeks after the campaign had ended, this says little about long term effects, and no observations and interviews were carried out after the end of the campaign. Finally, this was a field study without the possibility of having a control group, which weakens the possibility to make causal inferences, also our interpretation is backed by the comparison to sales data from the same period the year before (where there was no campaign).

## Conclusions

Overall, the results contain some encouraging signs that the Keyhole campaign indeed had impacts on shopping behaviour, and that it is possible to address target groups not usually responsive to campaigns on healthier eating, if the campaign is especially designed to address this target group. While this study is specifically on the Keyhole, the results have implications for the promotion of front-of-pack health symbols in general. Apart from their attention getting properties, the use of health symbols is highly dependent on top-down processes linked to the salience of the health motive at the point of purchase, and our results suggest that a multi-channel campaign can make the health motive more salient, at least for consumer groups where the base rate of health motive salience is low. Further research in this area should address more long-term effects, and should ideally involve a design where transaction data can be linked to individual data.
